# Utility of transoral pharyngeal ultrasonography for puncture drainage of peritonsillar abscess

**DOI:** 10.1002/ccr3.2032

**Published:** 2019-02-19

**Authors:** Yuta Hagiwara, Takashi Araga, Yoshimitsu Saito, Yoshiyuki Sasano, Yuki Tamura, Takahiro Shimizu, Yasuhiro Hasegawa

**Affiliations:** ^1^ Department of Neurology St. Marianna University School of Medicine Kawasaki Japan; ^2^ Department of Otorhinolaryngology St. Marianna University School of Medicine Kawasaki Japan

**Keywords:** peritonsillar abscess, puncture drainage, transoral ultrasonography

## Abstract

Puncture drainage is usually needed to treat peritonsillar abscess. However, inadvertent carotid artery puncture may result in devastating complications. Preoperative transoral carotid ultrasonography (TOCU) is useful to delineate the anatomical relationship between the abscess and carotid artery. We present a case of peritonsillar abscess illustrating the utility of TOPU for safe drainage.

A 35‐year‐old man was admitted to our hospital because of pharyngalgia with fever. A peritonsillar abscess was detected on contrast‐enhanced computed tomography (Figure 1). Transoral ultrasonography revealed the location of the abscess and arteries, and image guidance was useful for puncture drainage of the abscess (Figure 2). Transoral ultrasonography is a well‐known technique for examining the carotid artery,^1^ as transoral carotid ultrasonography (TOCU). Transoral ultrasonography is also useful in otolaryngology, not only angiology.^2^ In transoral ultrasonography, a transvaginal probe is inserted into the mouth and pressed against the wall of the pharynx. For otolaryngology, we have termed this technique TOPU to distinguish it from TOCU. The oral space is narrowed when the probe is inserted into the mouth, so the puncture needle cannot be used during TOPU. We plan to develop an attachment to the probe to allow ultrasonographic guidance of puncture drainage in the future. We determined the direction and depth of puncture based on the information of TOPU imaging. The drainage was successful, and the patient had a good clinical course. Although mis‐puncture of an artery represents a serious complication in the drainage of peritonsillar abscess, TOPU improves the safety of drainage.

**Figure 1:**
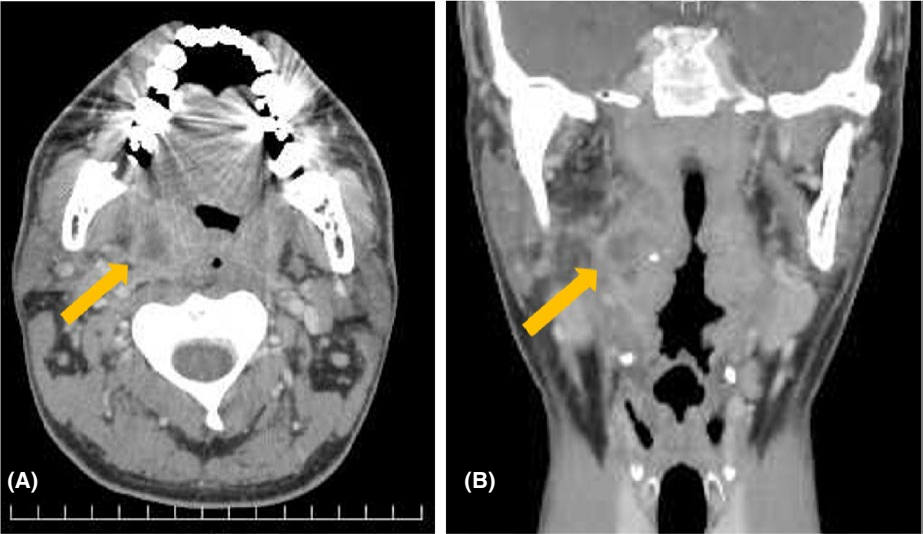
Contrast‐enhanced CT. Yellow arrow indicates peritonsillar abscess. A, Axial view; B, Coronal view

**Figure 2:**
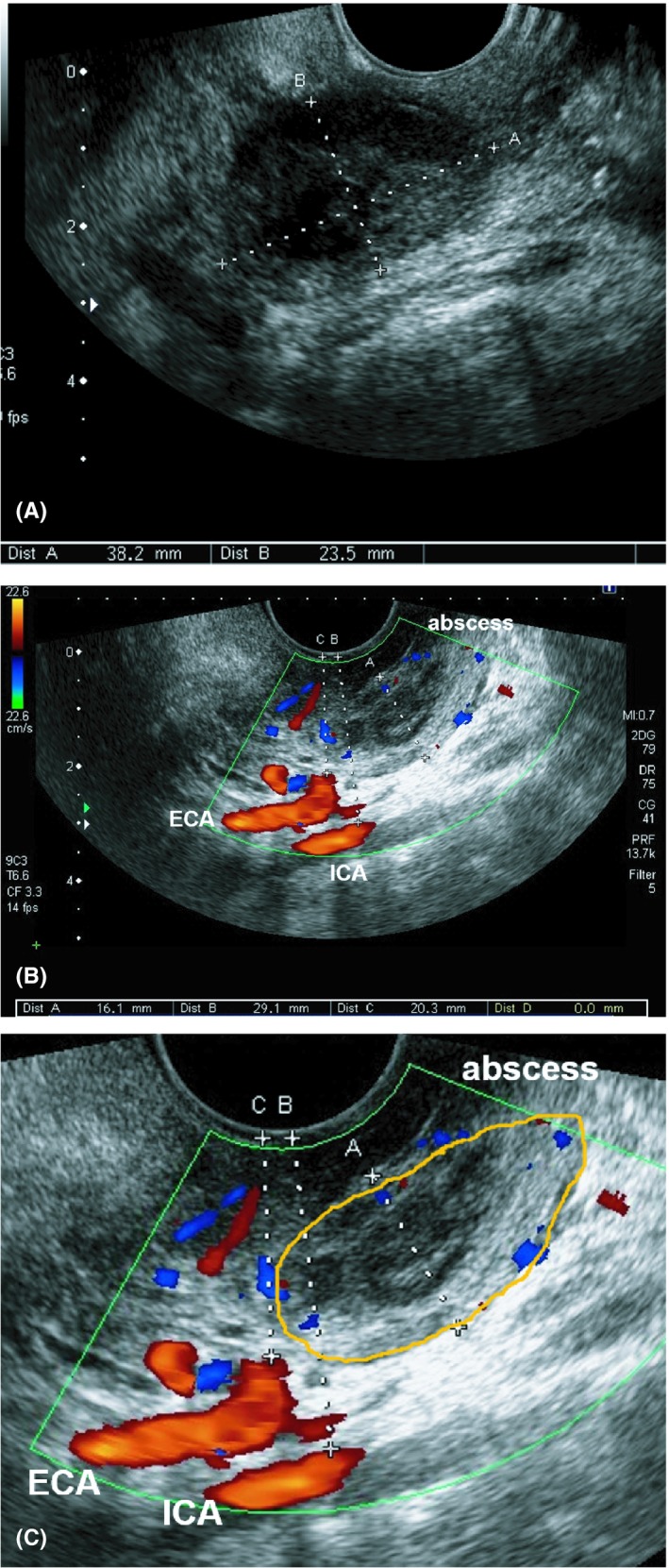
Transoral pharyngeal ultrasonography (TOPU). Aplio500 (Canon Medical Co., Tochigi, Japan) was used in ultrasound examination. 6 MHz transvaginal probe was used for TOPU. The patient was placed sitting position during TOPU. We did not use pharyngeal anesthesia. The probe is inserted into the mouth while avoiding the tongue and pressed softly against the wall of the pharynx in TOPU. A, TOPU with B‐mode. TOPU reveals a 38.2 mm × 23.5 mm abscess. B, TOPU with color Doppler (long axis). Right side of image is head side, and left side is foot side. TOPU reveals the internal carotid artery (ICA) and external carotid artery (ECA) just under the abscess at a depth of 20.3 mm. C, Schematic image. Yellow line indicates the periphery of the abscess

## CONFLICT OF INTEREST

None of the authors have any disclosures to report.

## AUTHOR CONTRIBUTION

YH: conceived the study and acquired, analyzed, and interpreted the data. TA, YS, YS, YT, and TS: acquired the data. YH: critically revised the manuscript for important intellectual content and supervised the study.
